# Supplementary material for: Production of recombinant human epidermal growth factor fused with HaloTag protein and characterisation of its biological functions
Source: PeerJ. 2024 Jul 16;12:e17806. doi: 10.7717/peerj.17806 (PMC11259126; doi:10.7717/peerj.17806)
Supplement: Supplemental Information 2 [file peerj-12-17806-s002.pdf]

Uncropped gel images for **Figure 1**

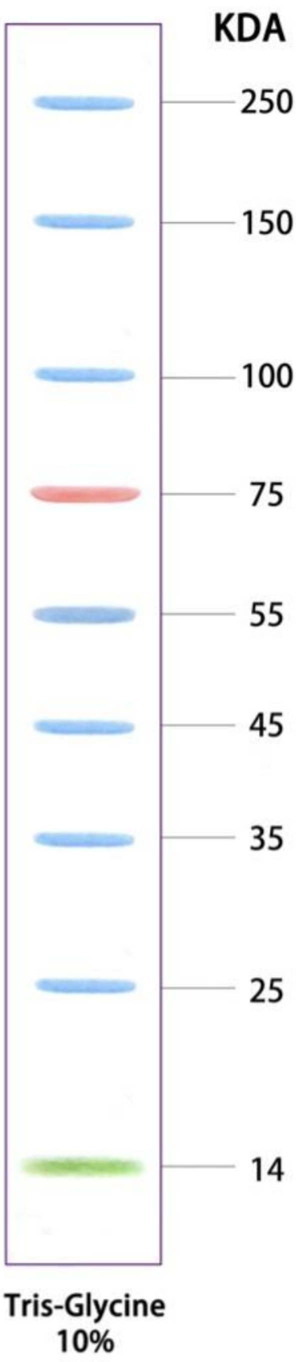

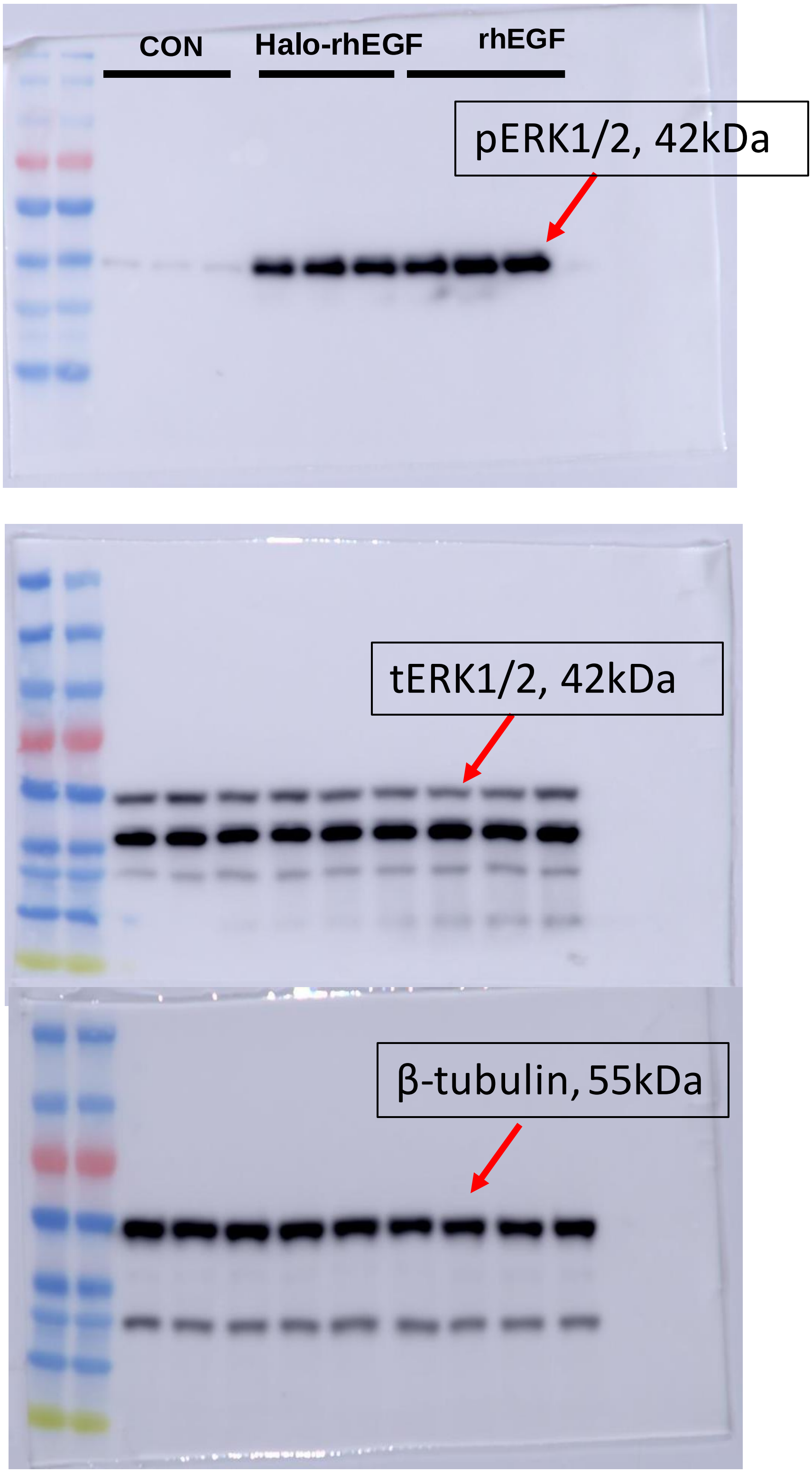

Uncropped gel images for **Figure 3**

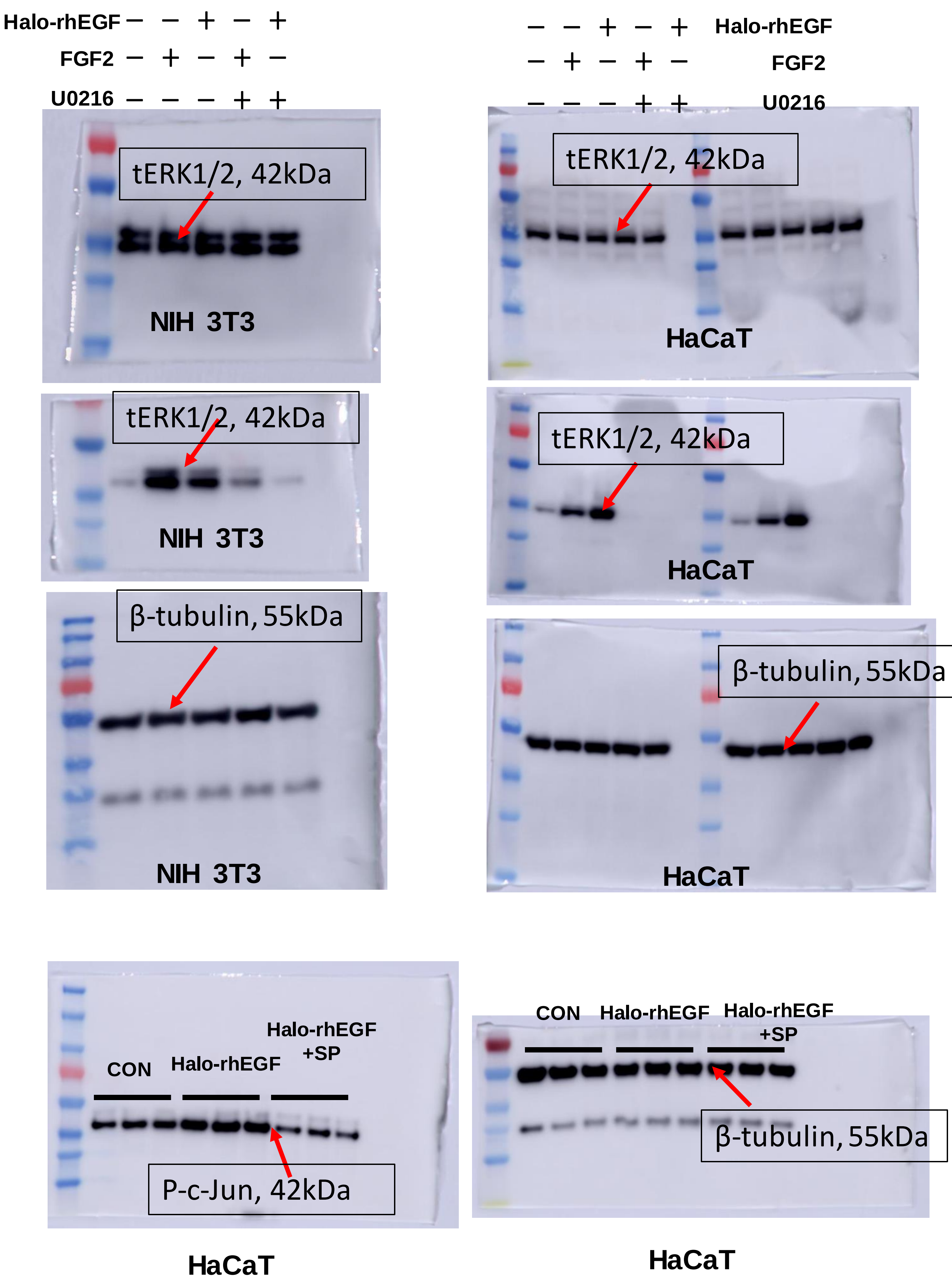

Uncropped gel images for **Figure 5**

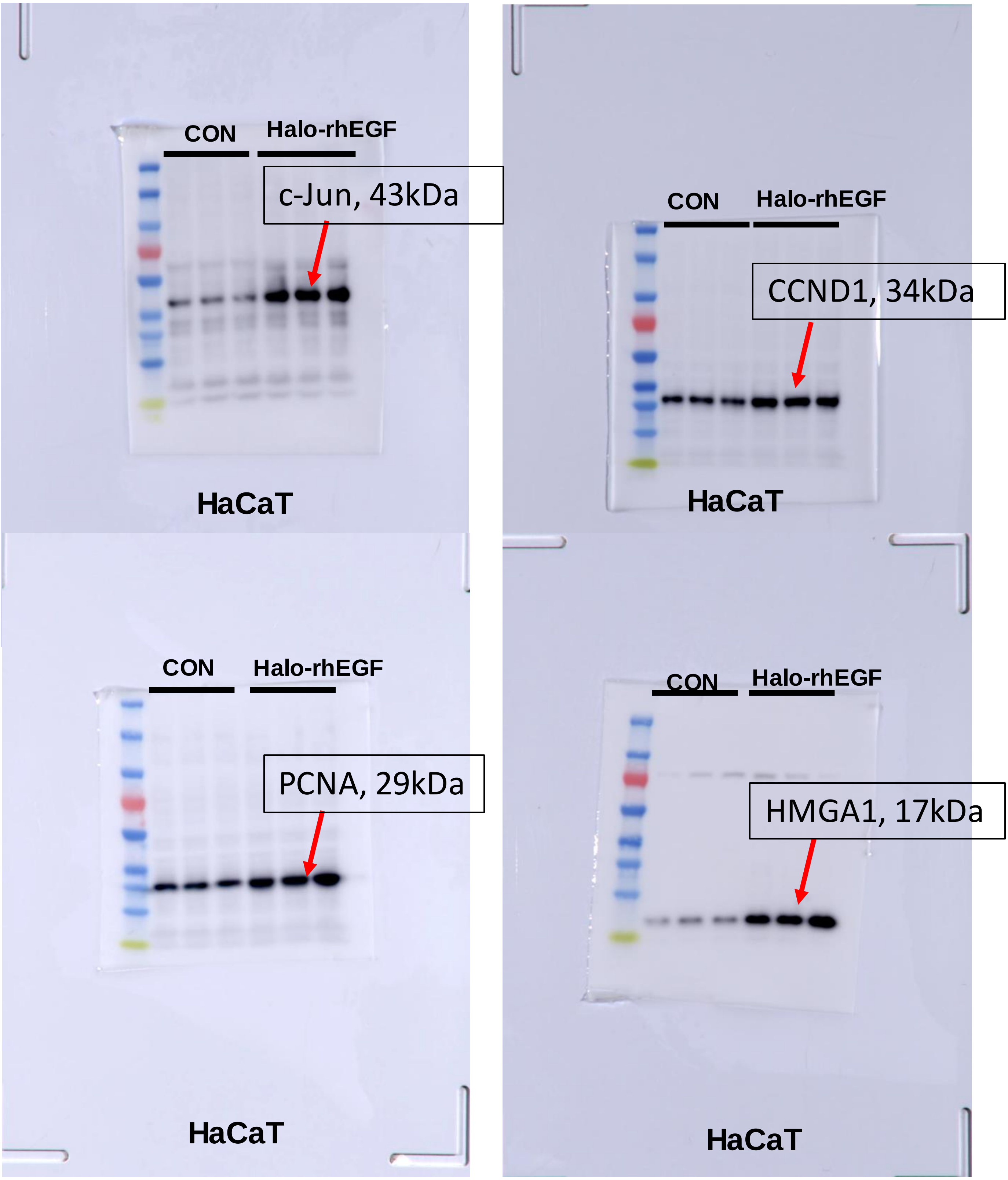

Uncropped gel images for **Figure 5**

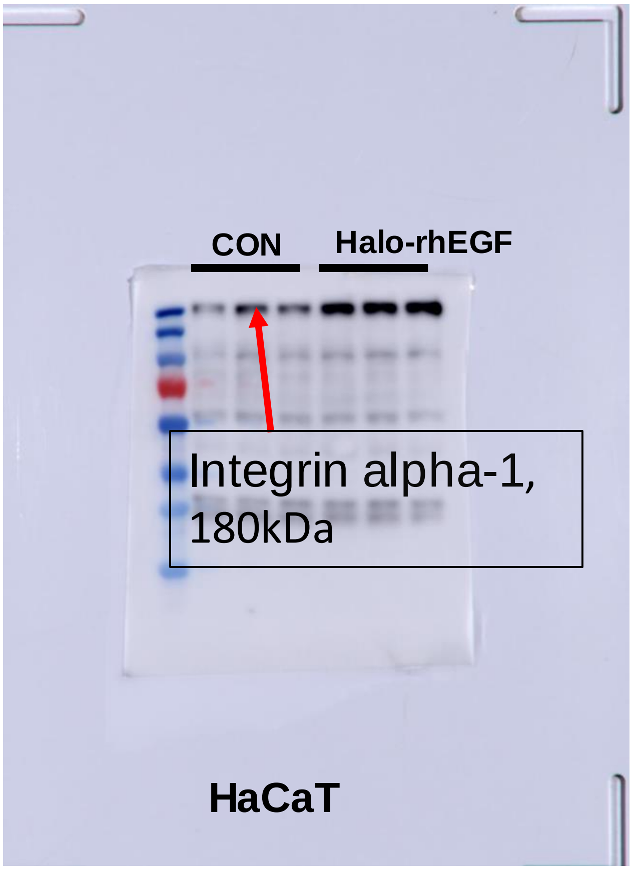

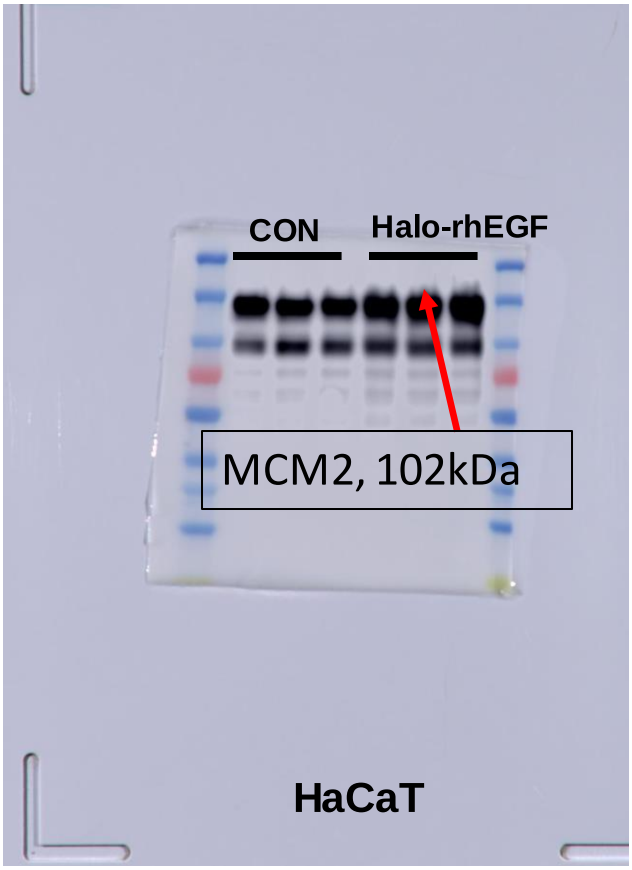

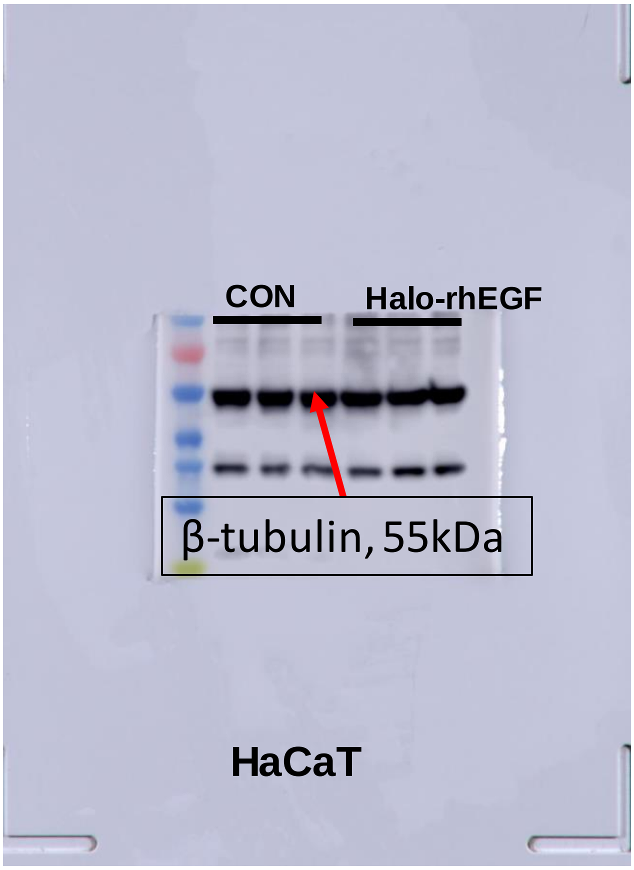

Uncropped gel images for **Figure 7**

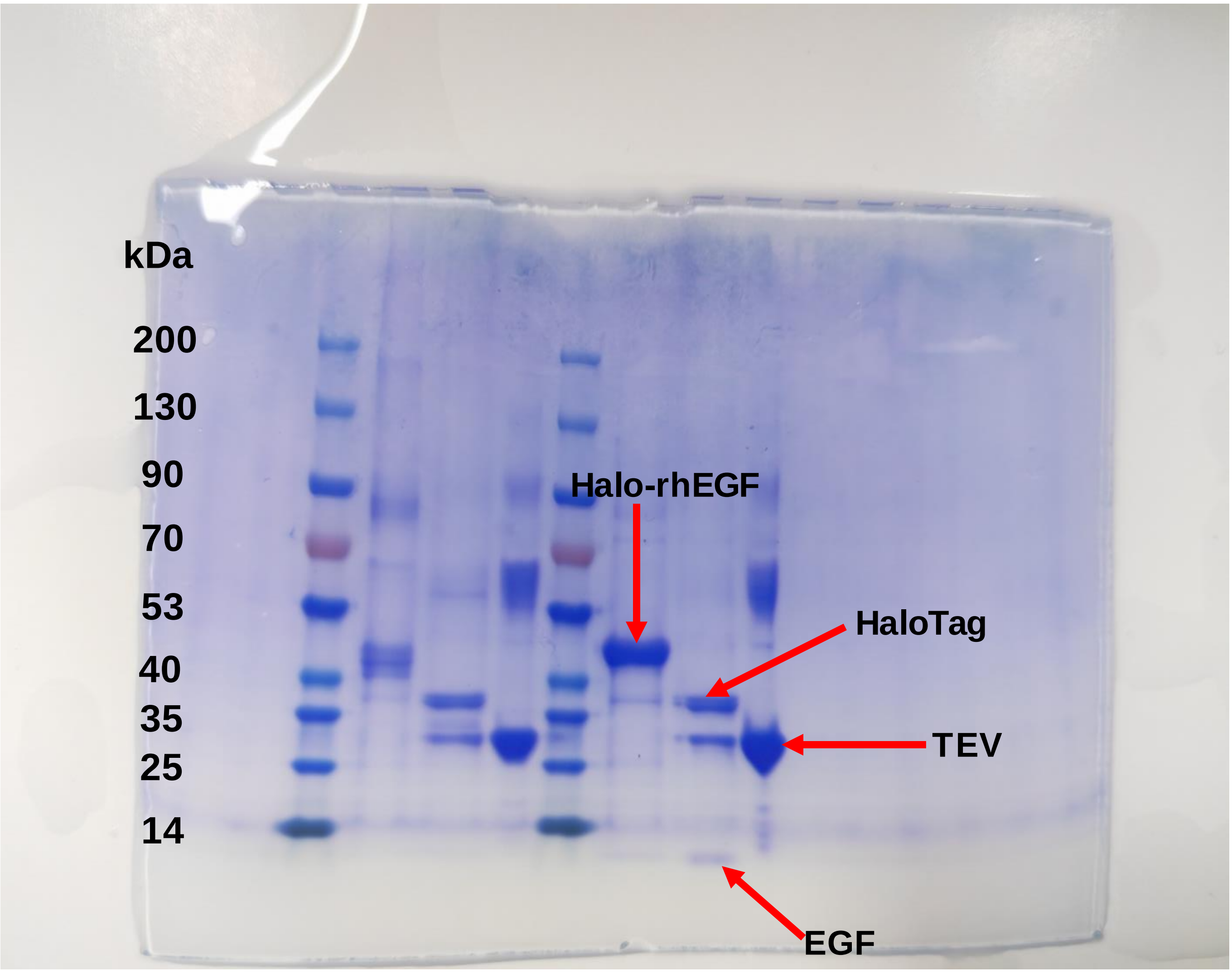

Coomassie Blue

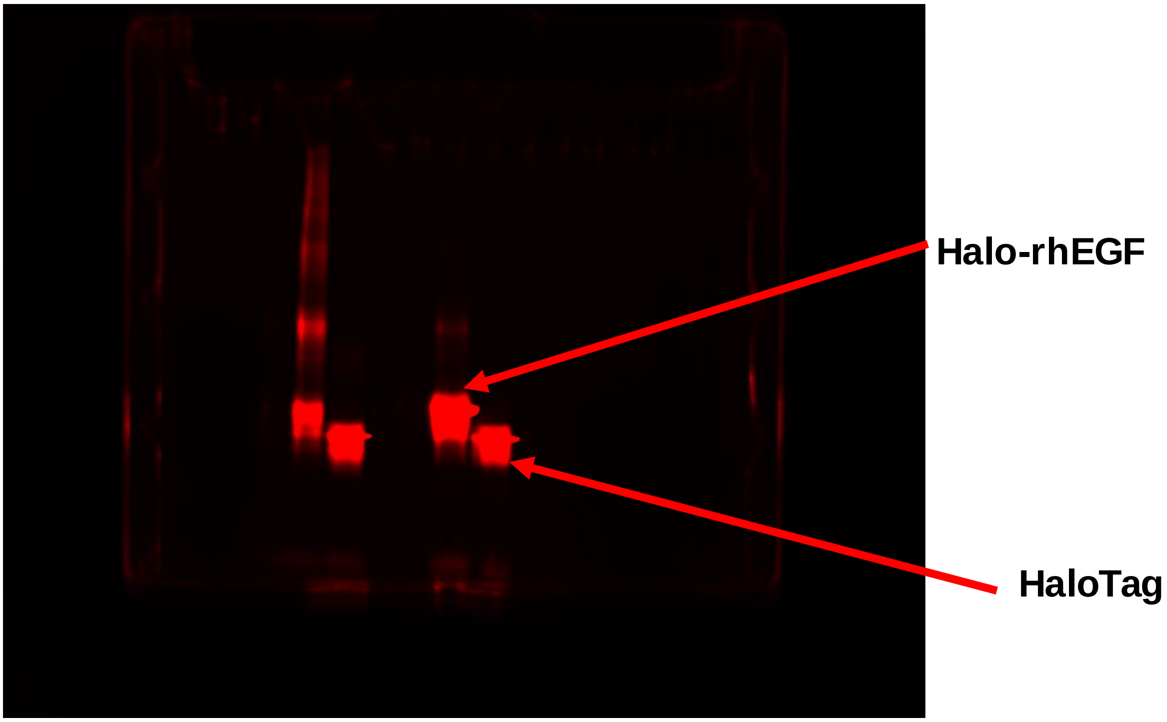

AlexaFluor 660 fluorescence
